# Olfactory Impairment Among Rural-Dwelling Chinese Older Adults: Prevalence and Associations With Demographic, Lifestyle, and Clinical Factors

**DOI:** 10.3389/fnagi.2021.621619

**Published:** 2021-04-12

**Authors:** Yi Dong, Yongxiang Wang, Keke Liu, Rui Liu, Shi Tang, Qinghua Zhang, Ingrid Ekström, Erika J. Laukka, Yifeng Du, Chengxuan Qiu

**Affiliations:** ^1^Department of Neurology, Shandong Provincial Hospital, Cheeloo College of Medicine, Shandong University, Jinan, China; ^2^Department of Neurology, Shandong Provincial Hospital Affiliated to Shandong First Medical University, Jinan, China; ^3^Aging Research Center and Center for Alzheimer Research, Department of Neurobiology, Care Sciences and Society, Karolinska Institutet-Stockholm University, Stockholm, Sweden

**Keywords:** olfactory impairment, hyposmia, anosmia, Sniffin' sticks identification test, old age, population-based study

## Abstract

**Objective:** Olfactory impairment (OI) refers to decreased (hyposmia) or absent (anosmia) ability to smell. We sought to estimate the prevalence and correlates of OI among rural-dwelling Chinese older adults.

**Methods:** This population-based cross-sectional analysis included 4,514 participants (age ≥65 years; 56.7% women) from the Multidomain Interventions to Delay Dementia and Disability in Rural China (MIND-China). The 16-item Sniffin' Sticks identification test (SSIT) was used to assess olfactory function. Olfactory impairment was defined as the SSIT score ≤10, hyposmia as SSIT score of 8–10, and anosmia as SSIT score <8. Multivariable logistic regression models were used to examine factors associated with OI.

**Results:** The overall prevalence was 67.7% for OI, 35.3% for hyposmia, and 32.5% for anosmia. The prevalence increased with age for OI and anosmia, but not for hyposmia. The multivariable-adjusted odds ratio (OR) of OI was 2.10 (95% CI 1.69–2.61) for illiteracy and 1.41 (1.18–1.70) for elementary school (vs. middle school or above), 1.30 (1.01–1.67) for current smoking (vs. never smoking), 0.86 (0.74–0.99) for overweight and 0.73 (0.61–0.87) for obesity (vs. normal weight), 4.21 (2.23–7.94) for dementia, 1.68 (1.23–2.30) for head injury, and 1.44 (1.14–1.83) for sinonasal disease. Illiteracy in combination with either male sex or diabetes was significantly associated with an over two-fold increased OR of OI (*p* for interactions <0.05).

**Conclusion:** Olfactory impairment is highly prevalent that affects over two-thirds of rural-dwelling older adults in China. OI is correlated with illiteracy, current smoking, dementia, head injury, and sinonasal disease, but negatively associated with overweight or obesity. Olfactory impairment as a potential clinical marker of neurodegenerative disorders among older adults deserves further investigation.

## Introduction

Olfactory impairment (OI) refers to a reduced (hyposmia) or absent (anosmia) ability to smell. Olfactory impairment has significant impact on everyday safety, quality of life, food preferences, and nutritional status (Attems et al., 2015), and is related to increased mortality among older adults (Liu et al., 2019). Olfactory function starts to decline approximately from the sixth decade of life (Kondo et al., 2020). Among older adults, the prevalence rates of OI vary substantially from ~20 to ~75%. For example, the U.S. Epidemiology of Hearing Loss Study found that the prevalence of impaired olfaction was 24.5% among participants aged 53 years or older (Murphy et al., 2002). Similarly, the U.S. National Health and Nutrition Examination Survey (NHANES) showed that 21.6% of people aged ≥60 years experienced impaired olfactory function (Hoffman et al., 2016). In addition, the Swedish National study on Aging and Care in Kungsholmen (SNAC-K) found that 24.8% of older adults (age ≥60 years) suffered from OI, with the prevalence being 19.1% for hyposmia and 5.7% for anosmia (Seubert et al., 2017). Moreover, in the Germany Dortmund Health Study OI affected nearly 30% of people aged 53 years or older (Vennemann et al., 2008). Other studies have reported relatively high prevalence of OI. For example, the Spain Olfaction in Catalonia survey reported that 60.7% of participants aged 60 years or older suffered from OI (57.9% hyposmia and 2.8% anosmia) (Mullol et al., 2012). In addition, the prevalence of OI was 74.4% in the Honolulu-Asia Aging Study of Japanese American men who were aged >70 years and free of clinical Parkinson's disease and dementia (Ross et al., 2008). It is well-established that odor identification is dependent on individual's familiarity with the odors and might be affected by cultural factors (Konstantinidis et al., 2008; Feng et al., 2019). Nevertheless, prevalence data of OI, hyposmia, or anosmia among Chinese older adults are still scarce.

Evidence has shown that sociodemographic (e.g., age, sex, and education), genetic (e.g., APOE ε4 allele), behavioral (e.g., smoking, physical inability), and metabolic factors (e.g., obesity, diabetes) are related to impaired olfaction among older people. Furthermore, OI may be correlated with clinical conditions, such as upper respiratory infections, sinonasal disease, and head trauma (Murphy et al., 2002; Vennemann et al., 2008; Boesveldt et al., 2011; Mullol et al., 2012; Pinto et al., 2014; Hoffman et al., 2016; Liu et al., 2016; Dong et al., 2017; Seubert et al., 2017; Stogbauer et al., 2020). Of note, previous studies have shown that OI is associated with Parkinson's disease and Alzheimer's disease (Dong et al., 2017; Doty, 2017), suggesting that OI may be a clinical marker that could be useful for early detection and clinical diagnosis of these neurodegenerative disorders. However, most of these previous studies have been conducted in high-income countries where people have relatively high educational attainment. In addition, factors correlated with OI may vary across socioeconomic and sociocultural contexts.

Therefore, in this population-based cross-sectional study, we sought to investigate the prevalence and a range of correlates of OI, hyposmia, and anosmia among rural-dwelling Chinese older adults.

## Materials and Methods

### Study Population

The study sample was from baseline participants in the Multidomain Interventions to Delay Dementia and Disability in Rural China (MIND-China) study, a participating project in the World-Wide FINGERS Network (Kivipelto et al., 2020). Briefly, MIND-China targeted people who were aged 60 years and older and living in the 52 villages of Yanlou Town, Yanggu County, western Shandong province, China. In March-September 2018, 5,765 individuals (57.2% women) were enrolled in the MIND-China study. Of these, 519 participants who were aged 60–64 years were excluded because they were highly selective owing to the fact that a majority of people in this age group were not available for the examination. Of the 5,246 participants who were aged 65 years and older, 732 had missing data on olfactory function due to refusal (*n* = 542) or incomplete olfactory test (*n* = 190), leaving 4,514 (86.0% of all the eligible participants) for the current analysis.

Compared to participants in the analytical sample (*n* = 4,514), those who were excluded (*n* = 732) due to missing data on olfactory tests were older (mean age, 75.5 vs. 71.1 years, *p* < 0.001) and less educated (years of formal schooling, 2.0 vs. 3.3, *p* < 0.001), but the two groups did not differ significantly in the distribution of sex (*p* = 0.08).

The MIND-China project was approved by Ethics Committee at Shandong Provincial Hospital affiliated to Shandong University in Jinan, Shandong Province. Written informed consent was obtained from all participants, or in the case of persons with severe cognitive impairment, from close relatives. Research within the MIND-China project has been conducted in accordance with the ethical principles for medical research involving human subjects expressed in the Declaration of Helsinki. MIND-China was registered in the Chinese Clinical Trial Registry (registration no.: ChiCTR1800017758).

### Data Collection and Assessments

Trained medical staff collected data via face-to-face interviews, clinical examinations, cognitive testing, and laboratory test following standard procedures. Data included sociodemographic features (e.g., age, sex, and education), genetic factors (APOE genotype), behavioral factors (e.g., smoking status, alcohol consumption, and physical inactivity), metabolic factors (e.g., hypertension, diabetes, lipids, and obesity), and clinical conditions [e.g., depressive symptoms, Parkinson's disease, cardiovascular disease (CVD), cancer, sinonasal disease, dementia, and head injury]. The Mini-Mental State Examination (MMSE) was administered to examine global cognition. We defined dementia following the Diagnostic and Statistical Manual of Mental Disorders, Fourth Edition (DSM-IV), criteria.

Educational level was categorized as “illiteracy,” “elementary school,” and “middle school or above.” After an overnight fast, peripheral blood samples were taken and apolipoprotein E (APOE) genotypes were determined using the multiple polymerase chain reaction. Apolipoprotein E genotype was dichotomized into carriers vs. non-carriers of ε4 allele. Smoking status was categorized as never, former, or current. Alcohol drinking was classified as no or occasional drinking and regular alcohol consumption (at least once a week during the past 12 months). Then, regular alcohol intake was further grouped into light-to-moderate (≤14 standard drinks/week for men and ≤7 standard drinks/week for women) and heavy (>14 standard drinks/week for men, >7 standard drinks/week for women) alcohol consumption according to the frequency and quantity of alcohol consumption per week (Jarvenpaa et al., 2005). Physical inactivity at leisure time was defined as participating less than once a week in any leisure-time physical activities. Current use of medications (e.g., blood pressure-lowering drugs, blood glucose-lowering drugs, and lipid-lowering drugs) was recorded based on self-report, and whenever possible, vials were checked to verify the report. All medications were classified and coded according to the Anatomical Therapeutic Chemical (ATC) classification system, as previously reported (Cong et al., 2020). Arterial blood pressure was measured on the right arm in a sitting position after a 5-min rest using an electronic blood pressure monitor (Omron HEM-7127J, Omron Corporation, Kyoto, Japan), with the cuff maintained at the heart level. Hypertension was defined as having blood pressure ≥140/90 mm Hg or current use of antihypertensive drugs (ATC codes C02, C03, and C07–C09). Fasting blood glucose and serum lipids were measured in local clinical laboratories according to standard protocols. Diabetes mellitus was defined as having a self-reported history of diabetes, or fasting blood glucose level ≥7.0 mmol/L, or use of glucose-lowering drugs, or insulin injection (ATC code A10). Dyslipidemia was diagnosed according to the 2016 Chinese Guideline for the Management of Dyslipidemia in Adults (Joint committee for guideline revision, 2018). Body mass index (BMI) was calculated as measured weight in kilograms divided by height in meters squared and was categorized as normal (<24 kg/m^2^), overweight (24–27.9 kg/m^2^), and obese (≥28 kg/m^2^) (Zhou and Cooperative Meta-Analysis Group of the Working Group on Obesity in China, 2002). Depressive symptoms were measured using the 15-item Geriatric Depression Scale (GDS-15, score range: 0–15) and presence of depressive symptoms was defined as a total GDS-15 score ≥5 (Underwood et al., 2013). Cardiovascular disease included coronary heart disease, myocardial infarction, heart failure, and stroke.

### Olfactory Function Assessments

We used the 16-item Sniffin' Sticks identification test (SSIT, Burghardt Messtechnik GmbH, Tinsdaler Weg 175, 22880 Wedel, Germany) (Hummel et al., 1997), which is a well-established and standardized test with high test-retest reliability to assess olfactory function (Croy et al., 2015). The original SSIT consists of 16 felt-tip pens with common odors (orange, leather, cinnamon, peppermint, banana, lemon, liquorice, turpentine, garlic, coffee, apple, clove, pineapple, rose, anise, and fish). Because local people were not familiar with cinnamon, turpentine, and coffee, the three odors in the original test were replaced with mushroom, soy sauce, and sesame oil, respectively. Mushroom is one of the odors in Sniffin' test of odor memory (Croy et al., 2015), and the other two odors were in the modified version of the SSIT in China (Shu and Yuan, 2008). The examiner asked the participant to smell the odor for 3–4 s, and then identify the correct odorant from four descriptors in a card presenting both the names and pictures of these four odors. One point was given for each correct answer, with a total score ranging from 0 to 16. We defined OI as a SSIT score ≤10, which was further classified into diminished olfactory function (i.e., hyposmia) if the SSIT score ranged 8–10 and functionally absent sense of smell (i.e., anosmia) if the SSIT score was <8 (Oleszkiewicz et al., 2019). In addition, a brief questionnaire was administered to collect the data on history of sinonasal disease, including rhinitis, rhinosinusitis, nasal polyps, and nasal surgery.

### Statistical Analysis

We presented mean (standard deviation, SD) for continuous variables and frequencies (%) for categorical variables. Characteristics of the study participants by olfactory function were compared using Kruskal-Wallis tests for continuous variables and Chi-square test for categorical variables. We reported the age- and sex-specific prevalence of OI, hyposmia, and anosmia. Then, we used binary logistic regression models to estimate odds ratio (OR) and 95% confidence interval (CI) of OI associated with various factors. Multinomial logistic regression models were used to examine the associations of hyposmia and anosmia with these factors. Statistical interactions of demographic features and APOE genotype with lifestyle and clinical variables on OI were assessed by simultaneously entering the independent variables and their cross-product term into the same model. We reported the main results from two models: model 1 was controlled for age, sex, and education; and model 2 was additionally controlled for all other factors. All analyses were performed using SAS version 9.4 (SAS Systems, Inc. Cary NC). Statistical significance was set at two-tailed *p* < 0.05.

## Results

### Characteristics of the Study Participants

Characteristics of the study population by olfactory function are summarized in Table 1. The mean age of the 4,514 participants was 71.1 (SD, 4.9; age range 65–93 years), 56.7% were women, and the average years of formal schooling was 3.3 years (SD, 3.5; range 0–19 years, 38.1% illiteracy). Compared with normosmia, participants with anosmia were older, more often women, less educated, had a lower BMI, and more likely to have a history of dementia, Parkinson's disease, head injury, and sinonasal disease (*p* < 0.05). These differences also existed between normosmia and hyposmia, except sex and a history of sinonasal disease or Parkinson's disease (Table 1).

**Table 1:** Characteristics of study participants by olfactory function.

**Characteristics^a^**	**Total sample,** ***n* = 4,514**	**Olfactory function**		
		**Normosmia**,	**Hyposmia**,	**Anosmia**,
		***n* = 1,456**	***n* = 1,593**	***n* = 1,465**
Age (years)	71.1 (4.9)	69.9 (4.1)	71.0 (4.7)^b^	72.4 (5.4)^b^^c^
Female sex	2,559 (56.7)	802 (55.1)	891 (55.9)	866 (59.1)^b^
**Education Level**				
Illiteracy	1,720 (38.1)	456 (31.3)	618 (38.8)^b^	646 (44.1)^b^^c^
Elementary school	2,015 (44.6)	683 (46.9)	716 (45.0)	616 (42.1)
Middle school or above	779 (17.3)	317 (21.8)	259 (16.3)	203 (13.9)
**GENETIC FACTORS**				
APOE ε4 allele carrier	704 (16.1)	226 (16.1)	269 (17.3)	209 (14.7)
**BEHAVIORAL FACTORS**				
**Smoking Status**				
Never	2,883 (63.9)	925 (63.6)	1,006 (63.2)	952 (65.0)
Former	680 (15.1)	227 (15.6)	252 (15.8)	201 (13.7)
Current	950 (21.1)	303 (20.8)	335 (21.0)	312 (21.3)
**Alcohol Drinking**				
No or occasional	3,398 (77.1)	1,067 (75.7)	1,197 (76.7)	1,134 (78.9)
Light to moderate	784 (17.8)	261 (18.5)	288 (18.5)	235 (16.4)
Heavy	224 (5.1)	81 (5.8)	75 (4.8)	68 (4.7)
Physical inactivity	1,510 (33.5)	500 (34.3)	527 (33.1)	483 (33.0)
**METABOLIC FACTORS**				
Hypertension	2,991 (66.8)	986 (68.4)	1,027 (65.0)	978 (67.3)
Diabetes	658 (14.6)	216 (14.8)	234 (14.7)	208 (14.2)
Dyslipidemia	1,084 (24.0)	355 (24.4)	374 (23.5)	355 (24.2)
**Body Mass Index (kg/m**^**2**^**)**				
Normal (<24)	1,839 (41.0)	526 (36.3)	658 (41.6)^b^	655 (44.9)^b^
Overweight (24–27.9)	1,752 (39.0)	585 (40.3)	611 (38.7)	556 (38.1)
Obesity (≥28)	898 (20.0)	339 (23.4)	312 (19.7)	247 (16.9)
**CLINICAL FACTORS**				
Dementia	142 (3.2)	11 (0.8)	42 (2.6)^b^	89 (6.1)^b^^c^
Depressive symptoms	449 (10.2)	141 (9.8)	144 (9.2)	164 (11.6)**^c^**
Parkinson's disease	33 (0.7)	5 (0.3)	13 (0.8)	15 (1.0)^b^
Cardiovascular disease	1,489 (33.0)	476 (32.7)	537 (33.7)	476 (32.5)
Cancer	61 (1.4)	16 (1.1)	23 (1.4)	22 (1.5)
**History of Head Injury**				
No	4,269 (94.7)	1,397 (96.2)	1,495 (94.0)^b^	1,377 (94.2)^b^
Yes	237 (5.3)	56 (3.9)	96 (6.0)	85 (5.8)
Non-traumatic	146 (3.2)	38 (2.6)	57 (3.6)	51 (3.5)
Traumatic	91 (2.0)	18 (1.2)	39 (2.5)	34 (2.3)
Sinonasal disease	409 (9.6)	112 (8.2)	146 (9.7)	151 (10.9)^b^
SSIT score	8.7 (3.2)	12.2 (1.2)	9.1 (0.8)^b^	5.0 (2.0)^b^^c^

*Data are mean (SD) or n (%); SSIT, Sniffin' Sticks Identification Test*.

a *The number of participants with missing values was 135 for APOE genotype, 1 for smoking, 108 for drinking, 38 for hypertension, 25 for body mass index, 101 for depressive symptom, 8 for head injury, and 252 for sinonasal disease. As a covariate in subsequent analyses, a dummy variable was created for each of the categorical variables to represent those with missing values*.

b *P < 0.05 for the comparison with normosmia*.

c *P < 0.05 for the comparison with hyposmia*.

### Prevalence and Distribution of OI, Hyposmia, and Anosmia

**Figure 1:**
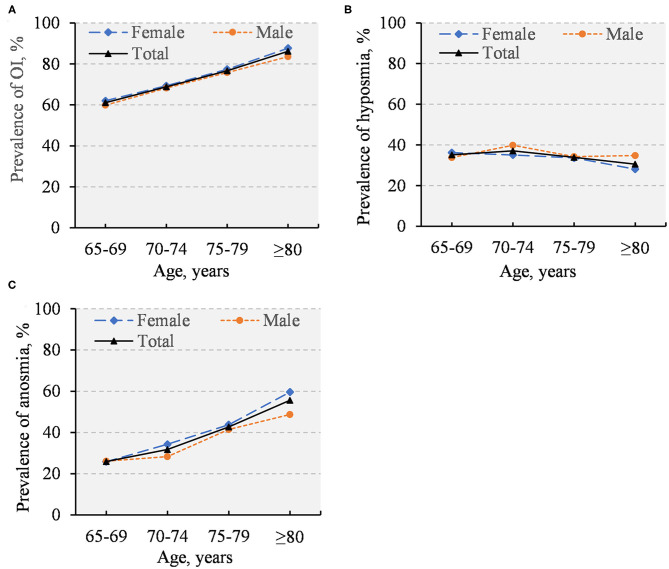
Prevalence of olfactory impairment **(A)**, hyposmia **(B)**, and anosmia **(C)** by age and sex (*n* = 4,514).

The overall prevalence of OI was 67.7% (95% CI: 66.4–69.1%), with the prevalence being 35.3% (33.9–36.7%) for hyposmia and 32.5% (31.1–33.8%) for anosmia. When people with dementia (*n* = 142) or Parkinson's disease (*n* = 33) were excluded, the overall prevalence was 66.8% for OI, 35.4% for hyposmia, and 31.4% for anosmia. The prevalence of OI and anosmia increased with advancing age, but the prevalence of hyposmia was relatively stable with age (Figure 1). Finally, there were no substantial sex differences in the prevalence of OI, hyposmia, and anosmia (Figure 1).

### Correlates of OI, Hyposmia, and Anosmia

Logistic regression analysis suggested that older age, male sex, and lower education were significantly associated with an increased likelihood of OI, hyposmia, and anosmia, but the association with sex became statistically non-significant in the multivariable-adjusted model (Table 2).

**Table 2:** Demographic, lifestyle, and clinical correlates of olfactory impairment, hyposmia, and anosmia (*n* = 4,514).

**Characteristics**	**Olfactory impairment**		**Hyposmia**		**Anosmia**	
	**Model 1^a^**	**Model 2^a^**	**Model 1^a^**	**Model 2^a^**	**Model 1^a^**	**Model 2^a^**
Age (years)	1.08 (1.07–1.10)^b^	1.08 (1.06–1.10)^b^	1.06 (1.04–1.07)^b^	1.05 (1.04–1.07)^b^	1.12 (1.10–1.13)^b^	1.11 (1.09–1.13)^b^
Sex (male vs. female)	1.23 (1.06–1.43)^b^	1.04 (0.82–1.33)	1.24 (1.05–1.47)^b^	1.10 (0.84–1.44)	1.21 (1.01–1.44)^b^	0.98 (0.74–1.30)
**Education Level**						
Illiteracy	2.15 (1.74–2.66)^b^	2.10 (1.69–2.61)^b^	1.93 (1.52–2.45)^b^	1.93 (1.51–2.46)^b^	2.43 (1.89–3.12)^b^	2.32 (1.79–3.00)^b^
Elementary school	1.41 (1.18–1.69)^b^	1.41 (1.18–1.70)^b^	1.36 (1.11–1.67)^b^	1.37 (1.12–1.69)^b^	1.48 (1.19–1.84)^b^	1.47 (1.18–1.84)^b^
Middle school or above	Reference	Reference	Reference	Reference	Reference	Reference
APOE ε4 carrier	1.02 (0.86–1.22)	1.00 (0.83–1.19)	1.11 (0.91–1.35)	1.09 (0.89–1.32)	0.92 (0.75–1.14)	0.89 (0.72–1.11)
**Smoking Status**						
Never	Reference	Reference	Reference	Reference	Reference	Reference
Former	1.14 (0.88–1.46)	1.12 (0.87–1.45)	1.14 (0.86–1.51)	1.12 (0.84–1.49)	1.12 (0.83–1.51)	1.12 (0.83–1.52)
Current	1.31 (1.03–1.66)^b^	1.30 (1.01–1.67)^b^	1.19 (0.91–1.55)	1.19 (0.90–1.57)	1.46 (1.10–1.94)^b^	1.45 (1.08–1.94)^b^
**Alcohol Drinking**						
No or occasional	Reference	Reference	Reference	Reference	Reference	Reference
Light to moderate	0.98 (0.81–1.19)	0.96 (0.79–1.17)	1.01 (0.81–1.25)	0.99 (0.80–1.24)	0.96 (0.76–1.20)	0.92 (0.73–1.17)
Heavy	0.97 (0.72–1.32)	0.93 (0.68–1.28)	0.90 (0.64–1.28)	0.89 (0.62–1.27)	1.06 (0.74–1.53)	0.99 (0.68–1.44)
Physical inactivity	1.02 (0.89–1.16)	0.99 (0.86–1.14)	0.99 (0.85–1.15)	0.98 (0.84–1.14)	1.05 (0.89–1.23)	1.01 (0.86–1.19)
Hypertension	0.85 (0.74–0.98)^b^	0.91 (0.79–1.04)	0.83 (0.71–0.97)^b^	0.87 (0.74–1.02)	0.88 (0.75–1.04)	0.95 (0.81–1.13)
Diabetes	1.06 (0.89–1.28)	1.10 (0.91–1.32)	1.06 (0.86–1.29)	1.09 (0.89–1.34)	1.09 (0.89–1.34)	1.11 (0.89–1.38)
Dyslipidemia	0.96 (0.83–1.12)	0.99 (0.85–1.16)	0.96 (0.81–1.14)	0.99 (0.83–1.18)	0.97 (0.81–1.16)	1.00 (0.83–1.20)
**BMI (kg/m**^**2**^**)**						
Normal (<24)	Reference	Reference	Reference	Reference	Reference	Reference
Overweight (24–27.9)	0.85 (0.73–0.98)^b^	0.86 (0.74–0.99)^b^	0.86 (0.73–1.02)	0.88 (0.74–1.03)	0.83 (0.70–0.98)^b^	0.84 (0.70–0.99)^b^
Obesity (≥28)	0.71 (0.60–0.84)^b^	0.73 (0.61–0.87)^b^	0.77 (0.63–0.93)^b^	0.79 (0.65–0.97)^b^	0.64 (0.52–0.79)^b^	0.66 (0.53–0.82)^b^
Dementia	4.41 (2.36–8.24)^b^	4.21 (2.23–7.94)^b^	2.99 (1.53–5.85)^b^	2.93 (1.48–5.80)^b^	5.94 (3.12–11.29)^b^	5.57 (2.90–10.70)^b^
Depressive symptoms	1.10 (0.89–1.36)	0.98 (0.79–1.23)	0.95 (0.74–1.22)	0.89 (0.69–1.14)	1.28 (1.00–1.64)^b^	1.11 (0.86–1.43)
Parkinson's disease	2.48 (0.95–6.52)	2.51 (0.94–6.67)	2.29 (0.81–6.47)	2.34 (0.82–6.68)	2.72 (0.96–7.65)	2.70 (0.94–7.76)
Cardiovascular disease	0.99 (0.87–1.14)	0.99 (0.86–1.14)	1.03 (0.89–1.20)	1.04 (0.89–1.22)	0.95 (0.81–1.11)	0.93 (0.78–1.09)
Cancer	1.50 (0.84–2.69)	1.48 (0.82–2.66)	1.42 (0.75–2.72)	1.38 (0.72–2.65)	1.60 (0.83–3.11)	1.61 (0.83–3.15)
**Head Injury**						
No	Reference	Reference	Reference	Reference	Reference	Reference
Yes	1.69 (1.24–2.31)^b^	1.68 (1.23–2.30)^b^	1.68 (1.19–2.36)^b^	1.68 (1.19–2.36)^b^	1.73 (1.21–2.47)^b^	1.68 (1.18–2.41)^b^
Non-traumatic	1.47 (1.00–2.15)^b^	1.46 (0.99–2.15)	1.46 (0.96–2.22)	1.45 (0.95–2.22)	1.48 (0.95–2.29)	1.46 (0.94–2.28)
Traumatic	2.16 (1.27–3.66)^b^	2.15 (1.27–3.65)^b^	2.15 (1.22–3.78)^b^	2.15 (1.21–3.79)^b^	2.17 (1.20–3.91)^b^	2.15 (1.19–3.89)^b^
Sinonasal disease	1.42 (1.13–1.79)^b^	1.44 (1.14–1.83)^b^	1.29 (1.00–1.68)	1.31 (1.01–1.71)^b^	1.58 (1.21–2.06)^b^	1.61 (1.23–2.11)^b^

*BMI, Body mass index*.

a *Data are odds ratio (95% confidence interval). Participants without olfactory impairment (n = 1,456) held as referent category for binary logistic regression model and multinomial logistic models. Model 1 was adjusted for age, sex, and education; and in model 2, additional adjustment was made for all the other factors included in the table*.

b *P < 0.05*.

After controlling for sociodemographic factors, current smoking, dementia, a history of head injury, and sinonasal disease were significantly associated with an increased likelihood for OI, hyposmia (except current smoking), and anosmia, while hypertension and overweight or obesity were linked with a decreased likelihood of OI, hyposmia, and anosmia (except hypertension) (Table 2, model 1). These associations remained significant in the multivariable-adjusted model, except that the association of hypertension with a reduced OR of OI and hyposmia was attenuated and became statistically non-significant (Table 2, model 2). In addition, presence of depressive symptoms was significantly related to an elevated OR of anosmia in model 1, but the association became non-significant in model 2 when additionally controlling for multiple potential confounding factors. Parkinson's disease was associated with an over two-fold increased likelihood of OI, hyposmia, and anosmia, but the association was not statistically significant. Of note, when head injury was divided into non-traumatic and traumatic, traumatic brain injury appeared to have a stronger association with higher OR of OI, hyposmia, and anosmia. Finally, we found no significant associations of olfactory function with APOE genotype, alcohol consumption, diabetes, dyslipidemia, CVD, and cancer (Table 2).

**Figure 2:**
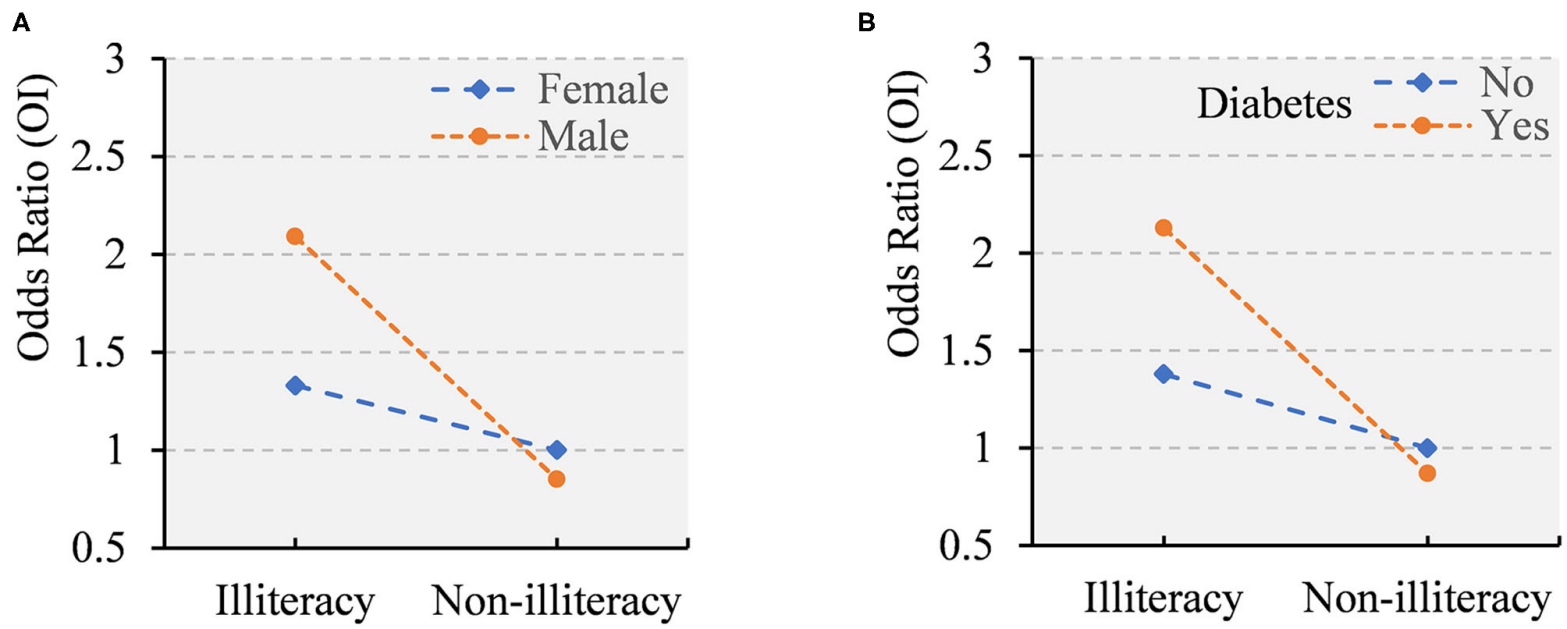
Interactions of educational level with sex **(A)** and diabetes **(B)** on olfactory impairment (OI) (*p* for both interactions <0.05).

We detected statistically significant interactions of illiteracy with sex and diabetes on OI (*p* for both interactions <0.05), such that illiteracy in combination with either male sex or diabetes was significantly associated with a substantially increased OR of OI (Figure 2). Supplemental Figure 1 shows joint effects of education (illiteracy vs. non-illiteracy) with either sex or diabetes on the likelihood of OI. Results from this analysis suggested that illiterate men (vs. non-illiterate women) had over two-fold increased OR of OI. Similarly, Illiterate persons with diabetes (vs. non-illiterate people without diabetes) had more than two-fold increased OR of OI.

We repeated the analyses after excluding participants with dementia and Parkinson's disease from the analytical sample, which yielded the similar results (data not shown).

## Discussion

We investigated the prevalence and correlating factors of OI, hyposmia, and anosmia among rural-dwelling Chinese older adults by using the standard olfactory identification assessment tool (SSIT). We found that OI was highly prevalent that affected more than two-thirds of older Chinese adults living in the rural communities. The prevalence of OI increased with advancing age, but there were no sex differences in the prevalence. Illiteracy, smoking, dementia, traumatic head injury, and sinonasal disease were correlated with an increased likelihood of OI, hyposmia, or anosmia, while overweight or obesity was linked to a decreased likelihood of olfactory dysfunction.

To the best of our knowledge, this is the first population-based study of OI that targets older adults living in the rural communities in China. Previous studies have shown that the prevalence of OI varies substantially across populations partly due to differences in methodological aspects (e.g., use of different olfactory function tests and cut-off values), sociodemographic characteristics of study populations (e.g., age, education, and race), and sociocultural factors. The overall prevalence of OI in our study population was similar to the report from a previous study in the USA, which showed that major olfactory impairment affected more than 60% of adults aged ≥65 years, and more than 75% of people aged ≥80 years (Doty et al., 1984). However, the prevalence of OI was much higher compared to the report from the Swedish SNAC-K study of highly educated urban residents (age ≥60 years) in central Stockholm (67.7 vs. 24.8%) (Seubert et al., 2017), where the similar 16-item Sniffin' Sticks test and the same cut-off scores were used to define OI. The sociocultural disparity (Konstantinidis et al., 2008; Feng et al., 2019) and the substantial differences in formal education (mean years of formal schooling: 12.4 in SNAC-K vs. 3.3 in MIND-China) and socioeconomic status might partly contribute to the differences in prevalence of OI. Indeed, odor identification is thought to involve high-level cognitive processes and as such likely to be influenced by educational level (Rahayel et al., 2012; Stogbauer et al., 2020). Low socioeconomic status has also been associated with OI (Schubert et al., 2012; Hoffman et al., 2016). Additionally, data from two U.S. large-scale studies of people aged ≥65 years showed that the overall prevalence of anosmia was 22.3% among blacks and 10.4% among whites (Dong et al., 2017), where the 12-item Sniffin' Sticks screening test and the 12-item Brief Smell Identification Test were used to assess olfactory function, and ~80% of the participants had a high school degree or above (vs. 4.5% in MIND-China cohort).

Our study showed decreased olfactory performance with advancing age, which is in accordance with the findings from several population-based studies of older adults (Murphy et al., 2002; Pinto et al., 2014; Dong et al., 2017; Seubert et al., 2017). Possible mechanisms include age-related alterations within the olfactory neuroepithelium (e.g., reduced number of olfactory sensory neurons), the olfactory bulb (e.g., atrophy of olfactory bulb), higher brain functional regions involved in olfactory processing (e.g., reduction in the volume of anterior olfactory nucleus, piriform cortex, amygdala, and hippocampus), and decreased neurogenesis with aging (Mobley et al., 2014; Attems et al., 2015). We did not observe sex differences in prevalence of OI, which was in contrast with previous studies that showed women outperformed men in olfactory function (Murphy et al., 2002; Boesveldt et al., 2011; Mullol et al., 2012; Stogbauer et al., 2020). A possible explanation could be that sex differences in olfactory function may decrease with increasing age (Larsson et al., 2004). In line with this explanation, a meta-analysis indicated that sex differences for odor identification existed only in younger adults (e.g., age 18–50 years), but not in older adults (e.g., age >50 years) (Wang et al., 2019a). It has been hypothesized that estrogens may enhance olfactory performance in women but estrogen levels decline markedly after the menopause (Doty and Cameron, 2009).

We also examined associations of several behavioral factors and clinical conditions with olfactory function. We observed an association of current smoking, but not former smoking, with poor smell identification ability, which was in accordance with the reports from some previous population-based studies (Murphy et al., 2002; Vennemann et al., 2008) and a meta-analysis (Ajmani et al., 2017), although other studies did not detect any association of smoking history with olfactory function (Liu et al., 2016; Dong et al., 2017). The possible mechanisms could be that the effects of smoking on the olfactory epithelium (e.g., squamous metaplasia and sinonasal inflammation) are reversible (Palmquist et al., 2020). Furthermore, we found that obesity was associated with better odor identification performance, which was similar to previous reports (Simchen et al., 2006; Dong et al., 2017). A possible explanation is that olfactory function, which appears to be necessary for the cephalic phase of digestion, affects food intake and dietary behaviors (Roberts and Rosenberg, 2006), which may be indirectly linked with a higher BMI. In support of this hypothesis, experimental research also found that mice with ablation of mature olfactory sensory neurons displayed a substantial reduction in body weight even under normal chow diet feeding conditions. Reduced olfactory input could stimulate sympathetic nerve activity and promote lipolysis (Riera et al., 2017). We found no association between diabetes and OI, however, there was a statistical interaction between illiteracy and diabetes on OI such that illiteracy in combination with diabetes was associated with an over two-fold increased OR of OI. The mechanisms of the interaction are not fully understood, but illiteracy and diabetes are known to be associated with cognitive impairment in older adults (Livingston et al., 2017; Wang et al., 2019b), which together may contribute synergistically to poor odor identification performance.

It has been reported that head trauma, nasal sinus disease, prior upper respiratory tract infection, and neurodegenerative disease (e.g., Parkinson's disease, and Alzheimer's disease) are four common causes of olfactory dysfunction in older adults (Murphy et al., 2002). Indeed, we found that sinonasal disease was associated with a higher likelihood of OI. It is generally agreed that inflammation, obstruction, reduced volume of olfactory bulb, and pathological changes in the olfactory mucosa caused by sinonasal diseases can lead to OI (Konstantinidis et al., 2010; Rombaux et al., 2016). Dementia was correlated with an increased likelihood of OI, but the association of impaired olfactory identification ability with Parkinson's disease was not statistically evident, largely due to limited power. Previous studies suggested that olfactory dysfunction may be a clinical, or even a preclinical, sign of neurological diseases such as Alzheimer's disease and Parkinson's disease (Dong et al., 2017; Doty, 2017). Differential disruption of a common primordial neuropathological substrate may contribute to varying degrees of olfactory dysfunction in the neurodegenerative diseases (Doty, 2017). Although previous research showed mixed findings with regard to the relationship between traumatic brain injury and impaired olfaction (Schofield et al., 2014), we found an association between head injury, especially traumatic brain injury, and an increased likelihood of OI. Traumatic brain injury causes additional neurological disturbances (Firsching, 2017), which may be linked with OI (Schofield and Doty, 2019).

We did not detect an association between APOE ε4 allele and OI, although their association has been previously reported in studies of American and European older adults (Olofsson et al., 2010; Larsson et al., 2016; Josefsson et al., 2017). It is worthwhile to further investigate whether the potential genetic susceptibility of olfactory function varies by ethnic or racial groups.

Main strengths of our study include the large sample of community-based rural-dwelling Chinese older adults. Furthermore, olfactory function was objectively assessed using a standardized test with high reliability. Our study also has limitations. First, the cross-sectional design of the study makes it impossible to draw causal inferences regarding relationships between OI and the examined factors. Second, the cross-sectional association between various factors and OI may be subject to selective survival, which usually leads to an underestimation of true associations. Third, we used only odor identification test (SSIT) to assess olfactory function, whereas adding additional tests of olfactory function, such as odor threshold and odor discrimination tests, may provide more thorough assessment of olfactory dysfunction (Lötsch et al., 2008). Finally, participants who did not complete the olfactory test were older and less educated than those included in the analytical sample. Thus, cautiousness is needed when generalizing our study findings to other populations, even other rural populations in China.

In summary, this population-based, cross-sectional study found that OI was highly prevalent in rural-dwelling older adults in China. In addition to older age and illiteracy, OI was correlated with current smoking, a lower BMI, dementia, head injury, and sinonasal disease. Follow-up studies will help identify the potential causal and modifiable lifestyle and clinical factors of OI, and thus, may contribute to the development of preventive and therapeutic interventions.

## Data Availability Statement

The raw data supporting the conclusions of this article will be made available by the authors, without undue reservation.

## Ethics Statement

The studies involving human participants were reviewed and approved by Ethics Committee at Shandong Provincial Hospital affiliated to Shandong University in Jinan, Shandong Province. The patients/participants provided their written informed consent to participate in this study.

## Author Contributions

YDo, YW, YDu, and CQ contributed to the study concept and design. YDo, YW, RL, ST, and QZ contributed to the acquisition of data. YDo and KL contributed to the data analysis. YDo contributed to the drafting of the manuscript. YDu and CQ contributed to the supervision. All authors contributed to the critical revision of the manuscript for important intellectual content, article, and approved the submitted version.

## Conflict of Interest

The authors declare that the research was conducted in the absence of any commercial or financial relationships that could be construed as a potential conflict of interest.
