# Supplementary material for: Olfactory Impairment Among Rural-Dwelling Chinese Older Adults: Prevalence and Associations With Demographic, Lifestyle, and Clinical Factors
Source: Front Aging Neurosci. 2021 Apr 12;13:621619. doi: 10.3389/fnagi.2021.621619 (PMC8072018; doi:10.3389/fnagi.2021.621619)
Supplement: Supplementary file 1 [file Table_1.DOCX]

Supplementary Material

# Supplementary Figure


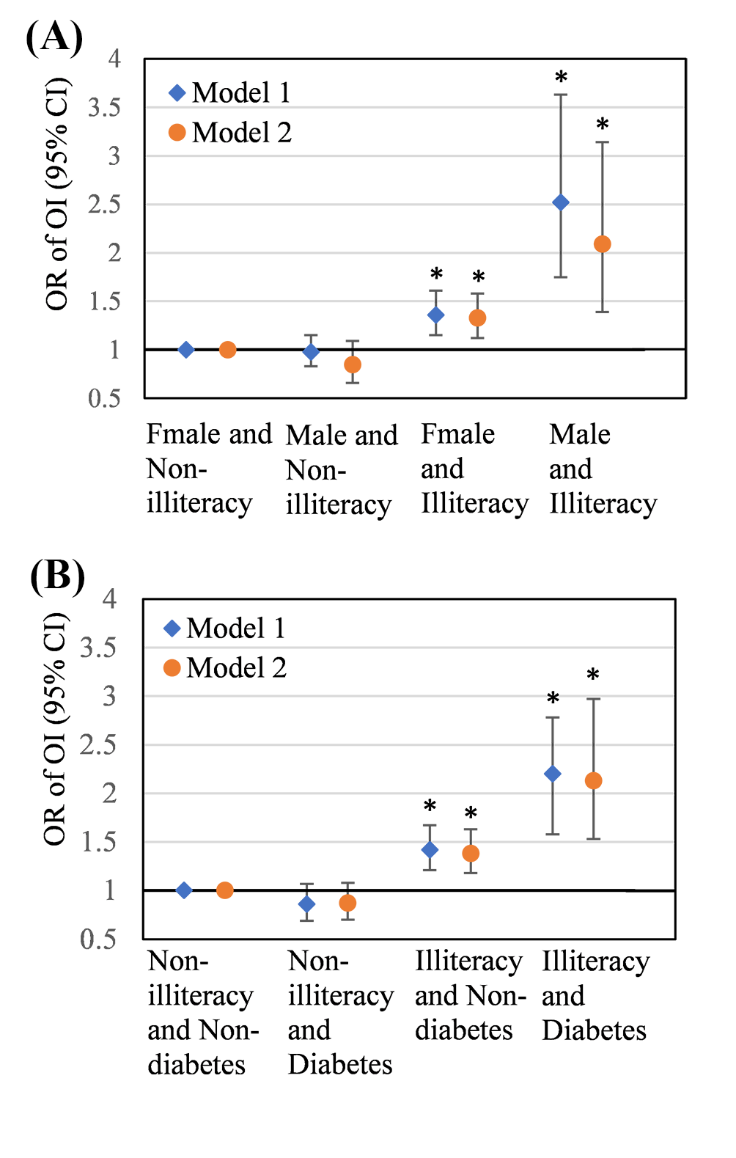


**Supplementary Figure 1.** Joint effects of illiteracy with male sex (A) and diabetes (B) on the odds ratio of olfactory impairment. *P<.05.

Model 1 was adjusted for age, sex, and education; and model 2 was further adjusted for smoking status, alcohol consumption, physical inactivity, hypertension, diabetes, dyslipidemia, obesity, depressive symptoms, Parkinson’s disease, cardiovascular disease, cancer, sinonasal disease, and head injury.

**OR, odds ratio; CI, confidence interval; OI, olfactory impairment.**
